# From devolution to post–COVID-19 UHC scoping review of human resource for health reforms in Kenya: a signalling and sensemaking analysis

**DOI:** 10.3389/frhs.2026.1786898

**Published:** 2026-07-01

**Authors:** Joseph O. Onyango, Jackline Oluoch-Aridi

**Affiliations:** 1 Centre for Social Behavior Change and Strrategic Foresight, Strathmore University, Nairobi, Kenya; 2 Centre for Social Behavior Change and Strrategic Foresight, University of Notre Dame, Nairobi, Kenya

**Keywords:** HRH-Kenya, post COVID-19, pre-devolution, sensemaking theory, signaling theory, UHC (universal health coverage)

## Abstract

**Background:**

Kenya's pursuit of Universal Health Coverage (UHC) has been profoundly shaped by health sector devolution 2010 and the COVID-19 pandemic both of which have exposed persistent challenges in human resources for health (HRH), including workforce shortages, geographic maldistribution, governance fragmentation, and declining motivation. While numerous studies have examined these challenges, less attention has been paid to how HRH reforms are interpreted by health workers and how policy signals shape workforce behaviour over time.

**Objectives:**

This scoping review synthesises evidence on HRH reforms in Kenya from 2000 to date (2025), examining the evolving HRH landscape, key barriers to recruitment, retention, and motivation, and documented interventions to strengthen workforce capacity. The review applies signalling and sensemaking theories to understand how HRH policies and reforms influence health worker perceptions, motivation, and retention.

**Methods:**

Following the Arksey and O'Malley framework and reported using PRISMA-ScR guidelines, we conducted a scoping review of peer-reviewed literature, policy documents, and grey literature published in English between 2000 and 2025. Searches were conducted across major bibliographic databases and institutional repositories. Sixty three sources met the inclusion criteria and were analysed using descriptive and thematic synthesis, guided by HRH and organisational theory frameworks.

**Findings:**

The evidence reveals a phased evolution of HRH reforms in Kenya, spanning pre-devolution, early devolution, maturing devolution, pandemic response, and post-COVID reform consolidation. Across these phases, inconsistent policy signalling—such as facility expansion without commensurate staffing, delayed remuneration, and fragmented governance—shaped health workers' sensemaking, often undermining motivation and retention, particularly in public and rural facilities. Conversely, credible signals, including digital health investments, task-sharing policies, strengthened community health systems, and leadership development initiatives, were associated with improved workforce engagement and performance.

**Conclusion:**

Kenya's HRH experience demonstrates that workforce reforms function not only as technical interventions but also as signals that health workers interpret through sensemaking processes. Aligning HRH policy design with clear, credible, and consistently implemented signals is critical to strengthening workforce motivation, retention, and performance in devolved health systems. These insights offer transferable lessons for other low- and middle-income countries pursuing UHC through decentralised governance.

## 1 Introduction

The landscape of human resources for health (HRH) in Kenya is characterised by various challenges and opportunities influenced by factors such as geographic distribution, staffing levels, training frameworks, and policy regulations across both public and private health sectors. This review synthesises current evidence related to HRH distribution, training, and policy frameworks, providing a foundational understanding of the HRH environment in Kenya. HRH landscape in Kenya illustrates significant progress amid challenges in workforce distribution, training, and policy implementation. Addressing these issues requires concerted efforts by stakeholders to enhance training quality, improve equity in workforce distribution, and implement effective policies to manage and support health workers in both the public and private sectors.

### 1.1 Overview of the landscape

The distribution of health workers across Kenya is uneven, with a pronounced concentration in urban areas relative to rural areas. Recent analyses indicate that private healthcare facilities generally have a more favourable staff distribution than their public counterparts, which tend to be significantly understaffed (1, 2). The public health sector is characterised by a paradox in which a significant percentage of health positions remain unfilled despite a larger operational workforce, pointing to inefficiencies in resource allocation. The Kenya National Health Workforce Strategy 2014–2018 indicated that approximately 14% of health workforce positions remain unfilled (3). Inadequate staffing undermines health service delivery, particularly in public facilities that serve a substantial portion of the population with limited financial resources (1). Moreover, geographic access to health services remains a critical issue. Research shows that effective geographic access to healthcare facilities is essential for good health service utilisation, necessitating ongoing assessments to address gaps in health infrastructure and service availability (4). The devolution of healthcare responsibilities after the promulgation of the 2010 constituion initiated in 2013, has influenced accessibility, with an aim to enhance local governance and improve service delivery at county and sub-county levels. However, disparities persist, especially in less populated rural areas where health worker shortages are pronounced (5).

Training and continuous professional development for health workers are crucial for enhancing workforce competency and service delivery. In Kenya, various training programmes exist, yet quality and consistency across facilities often vary. Evidence indicates that training improves health workers' performance, yet access to continuous professional development remains unequal (6). The absence of standardised training modules and disparities in resource allocation can hinder the growth and effectiveness of healthcare providers (7). Programmes aimed at skill enhancement and task-sharing, such as the National Task Sharing Policy, seek to optimise the distribution of health responsibilities among diverse health workers (8). Nonetheless, policy-level interventions are urgently needed to ensure that training is equitable and meets the healthcare system's demands, particularly in high-demand areas such as maternal and child health services (9).

The Kenyan healthcare system is governed by a complex array of policies that shape HRH management and practices. The most recent health policies are the second Kenya National Human Resource for Health Strategic plan 2014–2018 and the broader Kenya Health Sector Strategic Plan (KHSSP) 2018–2023 recognized Human Resources for Health (HRH) as a critical investment, aiming to address workforce gaps through defined strategies, norms, and standards for recruitment, retention, and efficient deployment, guided by the Human Resources for Health Strategic Plan and Devolved HRH Management policy to achieve Universal Health Coverage (UHC) goals despite resource constraints. Additionally, Kenya's Vision 2030 strategy emphasises the importance of robust health infrastructure and comprehensive workforce planning to achieve UHC (2). However, all these national policies often face implementation challenges, including industrial action that disrupts service delivery, as evidenced by various strikes affecting healthcare services (10, 11). Governance issues related to healthcare worker rights and working conditions have also attracted attention. The devolved government system has restructured health service delivery; however, it has also created challenges in ensuring accountability and performance within the health sector, particularly regarding the conditions of health workers and management practices (12). High absenteeism among health workers is exacerbated during industrial action, highlighting the need for better engagement and support systems (13).

### 1.2 Key HRH challenges and barriers in Kenya

This scoping review aims to elucidate the key barriers to workforce stability and performance. A prominent challenge in the Kenyan healthcare system is low job satisfaction and motivation among health workers. Evidence indicates that low salaries, delayed payments, inadequate professional development opportunities, and a lack of career advancement prospects substantially undermine job satisfaction (14, 15). Studies suggest that health professionals are increasingly disillusioned with their working conditions, resulting in high attrition rates, particularly in public health care settings (2, 16). Additionally, inadequate recognition of healthcare workers significantly affects their morale, prompting them to consider alternative employment opportunities in non-healthcare sectors or abroad (14).

Furthermore, the growth of the private health sector has intensified competition for skilled personnel, exacerbating retention issues in public hospitals, where working conditions are often less attractive (15). Consequently, the public sector faces the dilemma of providing essential health services with a demoralised and depleted workforce (16). The government's inability to fund competitive compensation packages further compounds these issues, as evidenced by widespread staff shortages and high turnover rates in key health areas such as nursing and community health (2, 3, 15).

Funding constraints significantly impede the recruitment and retention of health workers in Kenya. The healthcare budget has historically been constrained, with public health expenditure constituting only 2% of GDP, markedly below the 5% threshold recommended for low- and middle-income countries (17). This financial strain often results in inadequate resource allocation for health facilities, hampering operational capacity, including staff recruitment and retention initiatives (5). Moreover, administrative inefficiencies in distributing budgetary resources at county level have been identified as a barrier, leading to unpredictable wage disbursements and restricting investments in workforce development (18, 19). These conditions are further exacerbated by frequent delays in the national treasury's release of funds, which disrupt essential services and discourage healthcare professionals from sustaining their careers amid such uncertainty (18).

Political instability and corruption have also been identified as significant barriers to the effectiveness of the HRH system. Following the devolution of health services in Kenya, the number of health facilities increased; however, this was accompanied by mismanagement and political patronage that undermined recruitment processes (14, 16). The politicisation of health appointments can lead to nepotism and a lack of transparency, further deterring skilled professionals from entering the healthcare sector (2). Additionally, existing gender disparities within the health workforce affect recruitment and retention. Women face unique barriers to professional advancement, including limited access to training opportunities and systemic inequities in the workplace. Initiatives to support women's participation in training that enables career advancement are positive steps; however, they remain underfunded and under-implemented (20).

Lastly, workplace conditions and infrastructure play a critical role in shaping the employee experience in the healthcare sector. Many health facilities in Kenya lack adequate infrastructure, contributing to unsatisfactory working conditions (15). The lack of essential medical supplies, poor sanitation, and insufficient staffing affect not only healthcare delivery but also employee morale. Health workers report feelings of inadequacy and helplessness, which can prompt them to leave these critical roles (16).

### 1.3 Innovative interventions and strategies to improve HRH

The healthcare sector in Kenya has faced persistent challenges in HRH, prompting innovative interventions and strategies to enhance workforce capacity, improve distribution, and boost performance. These initiatives encompass the development of digital health solutions, task-sharing policies, community health engagement, and comprehensive policy reforms to address barriers to healthcare access and quality. One notable innovation is the adoption of digital health interventions, including mobile applications designed to enhance adherence to treatment protocols. For example, a study examined the feasibility of a health worker-targeted smartphone app to support the implementation of quality-assured rapid malaria diagnostic tests in Busia County. The findings indicated that the app was well received by health workers, thereby improving adherence to malaria treatment guidelines and highlighting the potential of digital solutions to enhance HRH performance (21). Community participation has been another key intervention, particularly in augmenting maternal and child health services. The Kenya community health strategy emphasizes the involvement of community health volunteers (CHVs), who provide essential services at the household level. Studies show that engaging CHVs significantly improves healthcare outreach and provides cost-effective maternal and child health services (22, 23). The development of policies that support community health structures has the potential to enhance healthcare delivery and reduce inequities in access (1, 22).

The implementation of task-sharing policies is a strategic response to HRH distribution disparities. The Kenyan government has introduced task-sharing guidelines to reallocate responsibilities among health workers, thereby optimising the existing workforce. This reallocation not only improves service delivery but also enhances health workers' morale by enabling better utilisation of skills across different cadres (24, 25). Documentation indicates that such models have been successfully used to meet primary health needs without placing undue pressure on specialised staff, demonstrating a flexible approach to HRH management (24). Additionally, the emphasis on mental health has gained prominence, with new mental health strategies being integrated into primary health care. Recognition of mental health as a critical component of health systems is reflected in ongoing training and resource allocation for healthcare providers to enhance their capacity to address these needs effectively (26, 27).

Despite these innovations, several challenges persist in implementing HRH strategies. Notably, bureaucratic inefficiencies, inadequate funding, and insufficient infrastructure often undermine the effectiveness of interventions. Funding gaps are particularly concerning, limiting the sustainability of successful initiatives (5, 17). Moreover, training and capacity-building efforts face barriers that are closely tied to HRH retention and motivation (2, 28). Conversely, opportunities exist for further integration of technology and collaborative practices. The potential for innovative partnerships between the public and private sectors, particularly in light of the COVID-19 pandemic, highlights a growing recognition of the need for collaborative strategies to enhance HRH capacity and performance (29, 30).

The impact of these innovative interventions on health outcomes is increasingly supported by empirical evidence. Enhanced community health engagement has been shown to improve maternal and child health outcomes, as evidenced by increased service utilisation (31). Furthermore, digital health innovations have been associated with improved adherence to treatment protocols, contributing to better health outcomes in supported areas (21, 32). However, realising these benefits fully depends on sustained funding, policy support, and infrastructure development to mitigate existing system barriers (2, 5, 17). While Kenya has made significant strides in innovative HRH strategies, maximising their benefits requires overcoming operational hurdles and fostering sustainable investment. Continued emphasis on evidence-based policy formulation and inclusive community engagement will further strengthen the healthcare workforce and enhance health outcomes nationwide.

### 1.4 Problem statement

The urgent need to strengthen HRH in Kenya is underscored by the ambition to achieve UHC. Despite several isolated studies identifying challenges and interventions in HRH, significant gaps remain in understanding the comprehensive landscape of workforce distribution, training systems, and policy frameworks. Addressing these gaps is essential to mapping the multifaceted issues that hinder health workers' stability, performance, and patient outcomes across both the public and private sectors. As Kenya grapples with critical challenges such as an inadequate workforce, geographic maldistribution, and insufficient training initiatives, these shortcomings pose substantial barriers to the realisation of UHC and to the overall health system's effectiveness. Ongoing deficiencies in financial allocations, organisational capacity, and infrastructural support exacerbate retention and motivation issues among health professionals (14, 15, 33). Furthermore, environmental challenges, including the impact of political dynamics and responses to public health emergencies such as COVID-19, underscore the complexities that influence the availability and functionality of health workers (14, 34).

The current moment is particularly pivotal for this scoping study, as the recognition of innovative strategies—such as digital health tools, task-shifting policies, and integrated health service delivery—presents a crucial opportunity to transform HRH capacity in Kenya (16, 35). While recent initiatives have shown promising results, such as enhanced adherence to treatment protocols through digital platforms and community engagement in healthcare delivery, a coherent framework that synthesises these approaches is necessary to scale up successful strategies (16, 36, 37).

Given the fragmented landscape of HRH research and the urgent demands of UHC, comprehensive exploration and documentation of existing and prospective interventions are vital. This scoping study aims to consolidate available knowledge, thereby providing evidence-based policy recommendations to optimise HRH strategies that can positively impact health outcomes across Kenya's diverse populations (38, 39). By identifying specific issues and leveraging documented solutions, the study aims to inform strategic actions that address ongoing workforce challenges in the healthcare system, ultimately contributing to a resilient and effective health workforce aligned with national and global health objectives. Overall, enhancing HRH capacity in the context of UHC in Kenya is an urgent imperative that requires concerted efforts and informed leadership to overcome systemic barriers and foster a sustainable health workforce ready to meet the population's health needs. This is achieved by postulating an empirical position, guided by the following research questions, in a consolidated manner.

*RQ1.* What is the current landscape of HRH in Kenya, including the distribution, training, and policy frameworks across health sectors?

*RQ2.* What are the key challenges and barriers affecting HRH reforms over the decade (2010–2024) in Kenya's healthcare sector?

*RQ3.* What innovative interventions and strategies have been documented to improve HRH capacity, distribution, and performance in Kenya?

## 2 Theoretical perspective

The intersection of signalling theory and sensemaking provides a nuanced lens for analysing the HRH landscape in Kenya. Challenges related to workforce capacity, distribution, and training are compounded by systemic financial and organisational barriers, affecting recruitment, retention, and motivation within the sector. Addressing these challenges through innovative strategies and policy reform requires effective signalling to both existing and prospective health workers, ultimately fostering a supportive environment conducive to health system strengthening in alignment with UHC objectives.

Signalling Theory holds that organisations send signals to convey their intentions or status to external stakeholders, including potential employees. In Kenya, the current landscape underscores a critical need for clear, robust signalling of the importance of health workforce capacity, geographic distribution, and training frameworks. Workforce size and distribution present significant gaps, particularly in rural areas, where health workers are less concentrated. Reports indicate that a high proportion of health professionals are based in urban centres, often neglecting rural populations reliant on public health systems for primary healthcare delivery (40). Training, education, and skills development are also critical areas where signalling can improve health workforce readiness.

Both the public and private health sectors require clarity on competency expectations; however, a cohesive strategy for workforce education in Kenya remains lacking (41). This inconsistency causes confusion among training institutions and prospective health workers about the competencies required in the evolving health landscape, particularly in managing digital health initiatives (42). Policy and regulatory frameworks further complicate the HRH landscape. Although Kenya has established policies to harmonise health workforce management, fragmented implementation often sends mixed signals about organisational priorities and commitments to health workforce development. This inconsistency can deter health workers from viewing the health sector as a rewarding career path (43).

Sensemaking Theory posits that individuals derive meaning from their experiences and interactions within an organisation. This theory is applicable to understanding the challenges that hinder recruitment, retention, and motivation in Kenya's healthcare sector. The experiences of health workers—shaped by financial insecurity, organisational policy vagaries, and workplace conditions—influence their perceptions and decisions regarding job satisfaction and career longevity. Retention, motivation, and job satisfaction are undermined by insufficient financial compensation and poor workplace conditions. Health workers often feel undervalued and overburdened due to inadequate resources and recognition, leading to high turnover rates (44). These experiences contribute to a negative sensemaking cycle where dissatisfaction begets attrition, further eroding the workforce. Funding, budgeting, and resource allocation are also crucial factors contributing to dissatisfaction in the health sector. Funding constraints limit organisations' ability to provide necessary incentives and training opportunities, leaving health workers feeling unsupported and demotivated (45). These financial barriers contribute to the interpretation of the organisational environment as hostile or unresponsive to employee needs. Workplace conditions and infrastructure directly impact health worker engagement. Poor infrastructure and inadequate facilities hinder daily operations, sending signals that the organization lacks commitment to maintaining a supportive work environment, thus negatively affecting perceptions of job security and fostering a sense of futility in the workforce (46).

To address these multifaceted HRH challenges, various innovative interventions and strategies have emerged. Signalling theory suggests that effective communication about these initiatives can enhance their acceptance and efficacy. For example, digital health initiatives represent a strategic response to improve access and efficiency within the healthcare system. These technologies not only enhance service delivery but also signal to health workers that the organisation is adapting to new realities and is invested in modernising its approaches to healthcare provision (47). Task-shifting policies also offer insights into how to adapt existing workforce capabilities to meet demands while maximising efficiency. By realigning responsibilities across healthcare cadres, organisations send a strong signal of adaptability that may alleviate some of the stress on the most specialised roles (48). Furthermore, comprehensive policy reforms rooted in evidence-based practices establish a foundation for continuous improvement and responsiveness in HRH management. The development of frameworks that define health workforce roles clearly can encourage better alignment of expectations and motivations among health professionals, fostering a more positive organisational climate (49).

## 3 Methods

This scoping review was conducted in accordance with the Arksey and O'Malley framework, refined by Levac et al. and reported using the PRISMA Extension for Scoping Reviews (PRISMA-ScR). A protocol was developed *a priori* to guide the study, ensuring rigorous methodological adherence and consistency throughout.

### 3.1 Eligibility criteria

We included empirical studies, policy documents, technical reports, and grey literature on HRH in Kenya, published in English between 2000 and 2025. Documents were eligible if they examined HRH distribution, training, policy frameworks, recruitment, retention, motivation, or interventions in the public and/or private health sectors. Clinical studies without an explicit HRH focus and papers lacking Kenya-specific data were excluded to maintain relevance and specificity within our focused scope.

### 3.2 Information sources and search strategy

A systematic search strategy was employed across multiple databases, including cross-reference databases, PubMed, Embase, Scopus, Web of Science, CINAHL, Global Health, and African Index Medicus. Supplementary searches were conducted using Google Scholar and the institutional repositories of Kenyan universities. The PubMed search strategy combined the terms “Kenya,” “health workforce,” and HRH concepts (e.g., recruitment, retention, training, and interventions) and was adapted for use in other databases.

### 3.3 Selection process

**Figure 1:**
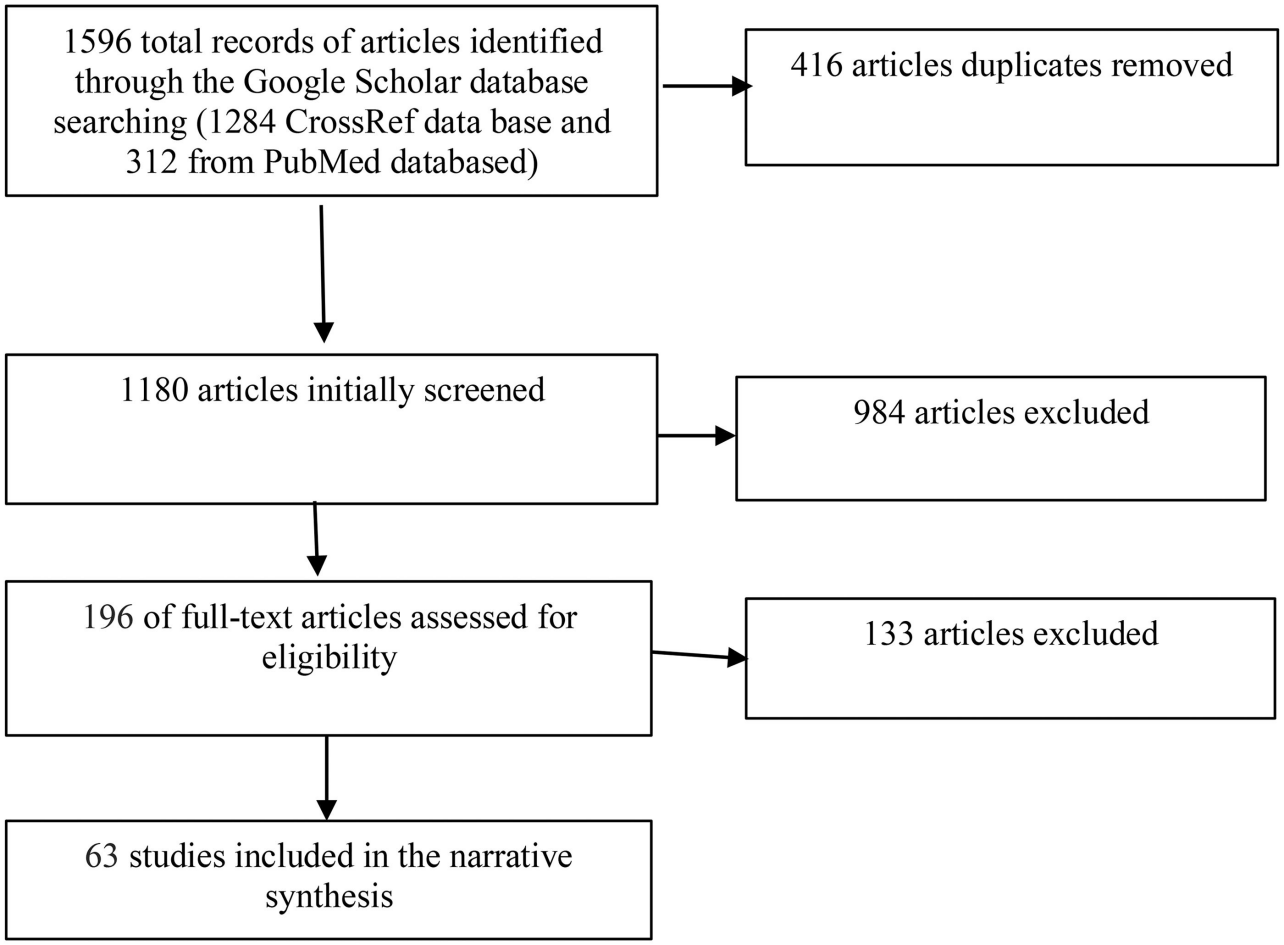
Study selection flow diagram summary.

All retrieved records were imported into Rayyan, a systematic review tool, for deduplication. Two independent reviewers screened titles and abstracts and later assessed full texts against the predetermined inclusion criteria. Disagreements on inclusion were resolved through discussion or by consulting a third reviewer, ensuring inter-rater reliability. Reasons for exclusion at each stage were documented, and the selection process is illustrated through a PRISMA flow diagram (Figure 1).

### 3.4 Data charting

A standardised form was developed to extract relevant study characteristics, including the author, year of publication, document type, specific HRH themes (distribution, training, policies, challenges), and documented interventions. Data extraction was performed by one reviewer and validated by a second reviewer to ensure accuracy.

### 3.4 Synthesis

The synthesis of results involved a descriptive numerical summary of included records categorised by year, type, cadre, and sector. A qualitative thematic analysis was used to interpret the findings, guided by established HRH frameworks. The results were systematically mapped across the three review questions: (1) the HRH landscape in Kenya, (2) barriers to recruitment, retention, and motivation, and (3) strategies and interventions to strengthen HRH. This comprehensive methodology aims to provide a thorough understanding of HRH in Kenya by identifying gaps, challenges, and opportunities for improving healthcare delivery.

We conducted a structured data-extraction and thematic analysis aligned to our research question on how health workforce features influence signalling and sense-making in Kenya's health system. From the provided reference list we extracted study characteristics (study type, population, setting, primary findings) and coded findings against an *a priori* coding framework derived from signalling and sense-making theory (signals: data, policies, financing, innovations; sense-making: leadership, governance, community interpretation). Coding proceeded iteratively: two reviewers independently coded all items, resolved discrepancies by discussion, and added inductive subthemes where novel patterns emerged. We mapped each coded item to the table themes (workforce distribution, training, governance, funding, innovations, retention, barriers, service impacts) and recorded supporting references. Triangulation across study designs and cross-checking of key claims with multiple sources were used to increase credibility. Limitations include reliance on published/available sources and heterogeneity of study designs.

## 4 Findings

### 4.1 Background information on available studies

The analysis of publications by year reveals a clear upward trajectory, starting with a single paper from 2010 and modest activity between 2012 and 2018, during which only two to four papers were published annually. Growth became more noticeable in 2019, with seven publications, and remained steady in 2020, with six, before accelerating sharply from 2021 onwards. This surge peaked in 2024, with 24 papers, making it the most productive year in the dataset. In 2025, six papers have been recorded so far, likely an incomplete count since the year is still ongoing. Overall, the trend suggests that research activity in this field has expanded significantly in recent years, with the last 5 years showing the strongest momentum and 2024 standing out as a landmark year.

The timeline shows a marked rise in HRH-related publications, with a notable peak in 2024. This reflects growing research attention to HRH issues, particularly following devolution and during and after the COVID-19 pandemic. Readers should note that year extraction was automated and may include non-primary-source years if cited (e.g., in data or policy documents).

**Figure 2:**
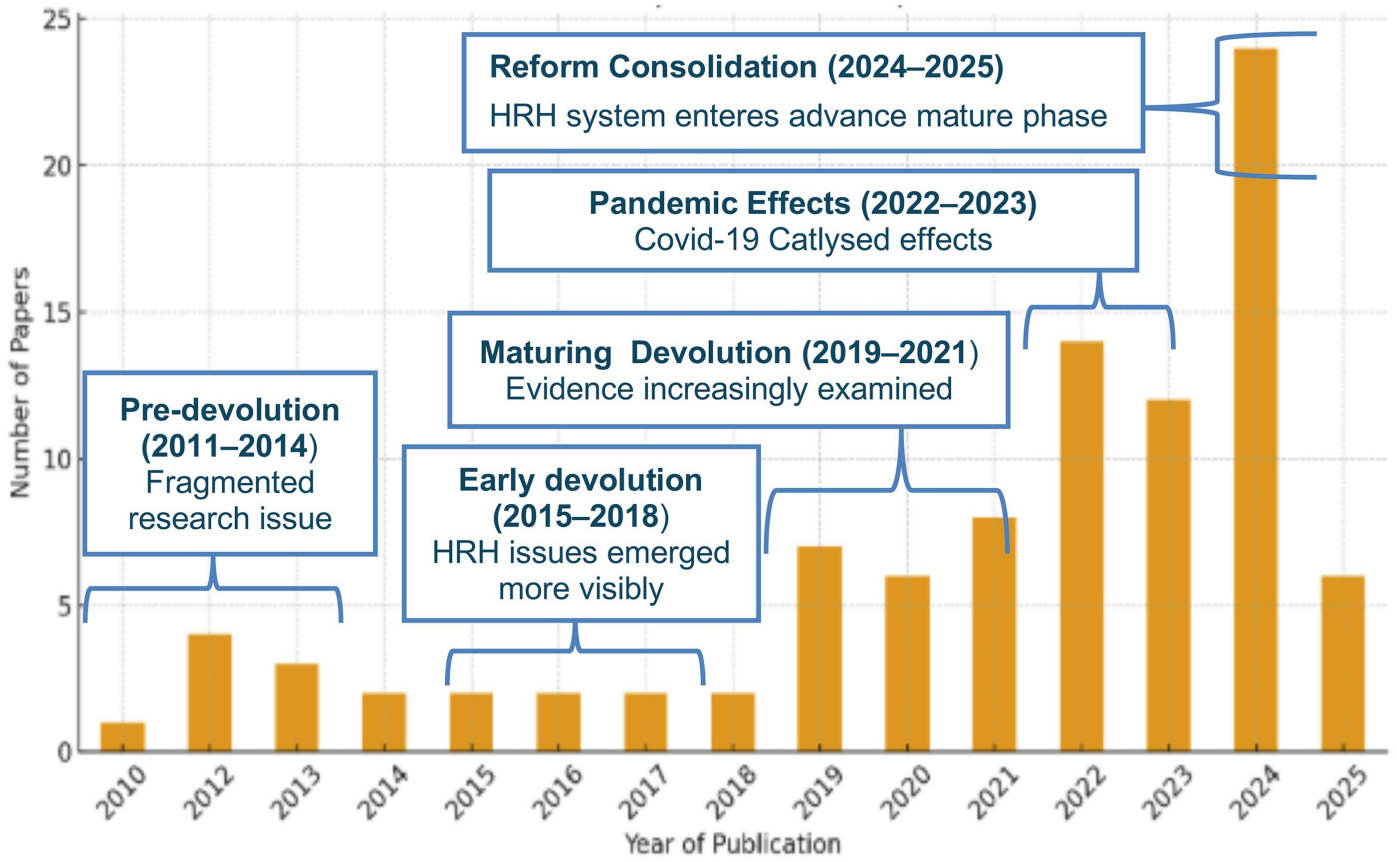
Emerging thematic focus over the years.

As illustrated in Figure 2, Kenya's HRH research trajectory shows a clear evolution across five phases. In the pre-devolution period (2011–2014), studies were largely fragmented, focusing on isolated issues and with minimal attention to HRH governance. During the early years of devolution (2015–2018), HRH challenges—particularly workforce distribution, shortages, and motivation—became more prominent in the evidence base. By the maturing devolution stage (2019–2021), research increasingly incorporated governance, leadership, and management dimensions of the health workforce. The pandemic period (2022–2023) acted as an accelerator, spurring research on surge capacity, digital health systems, CHV resilience, and task-sharing innovations. Entering the reform consolidation phase (2024–2025), Kenya's HRH system transitioned towards advanced modelling, policy experimentation, multi-sectoral collaborations with public and digital partners, and deeper professionalisation of the health workforce.

A further analysis of these findings demonstrates the effect of signalling and sense-making perspectives of the existing empirical evidence. Table 1 synthesises the reviewed studies into a phased interpretation of Kenya's HRH reforms through the lenses of signalling theory and sensemaking. The analysis demonstrates a progressive intensification of policy signalling and a corresponding evolution in frontline interpretive responses.

**Table 1:** Synthesis of the evolution from the periods in the lense of signaling and sensemaking.

Period	Intervention type	Core reform focus	Signalling	Sensemaking (frontline level)	Key references
Pre-devolution (2011–2014)	HRH capacity baseline & workforce gaps	Workforce shortages, maldistribution, weak planning	Weak institutional signalling; limited credible long-term workforce commitments	Workers interpret instability as limited career progression → defensive professional behaviour	(2)
	Service delivery fragility	Maternal health and service access constraints	Structural scarcity signals system vulnerability	Sensemaking framed around resource improvisation and coping	(9–11)
	Research & governance foundations	Health research and governance structures	Early normative signalling around reform need	Emerging professional identity formation	(39)
Early devolution (2015–2018)	County-level HRH governance	Decentralised planning and budgeting	Strong policy signalling of local autonomy; mixed consistency across counties	Ambiguity in role expectations; variation in trust toward county leadership	(14)
	Workforce distribution & equity	Geographic access disparities	Equity signalling through redistribution rhetoric	Perceived inequity fuels morale differences between urban/rural cadres	(4, 5)
	Motivation & work environment	Workplace climate and organisational culture	Relational signalling (management–staff interaction quality)	Positive leadership cues increase engagement; negative cues heighten exit intentions	(50, 51)
	Task-sharing frameworks	Formalised delegation policies	Competency signalling and scope-of-practice clarification	Expanded identity among mid-level cadres; legitimacy negotiation	(8, 24)
Maturing devolution (2019–2021)	HRH management & efficiency	Workforce efficiency and management reforms	Performance signalling and accountability cues	Increased managerial rationality; performance monitoring internalised	(16)
	Strike & stability effects	Workforce unrest and service disruption	Contradictory signalling: reform rhetoric vs. delayed remuneration	Erosion of trust; collective mobilisation behaviour	(9–11)
	Leadership & sustainability	Facility-level governance and leadership	Strategic signalling of sustainability and reform continuity Leaders frame reforms as long-term institutional strengthening		(35)
	Training & skill development	Clinical knowledge strengthening	Human capital investment signalling	Enhanced professional self-efficacy and retention intent	(7, 52)
Pandemic acceleration (2022–2023)	COVID-19 workforce response	Surge capacity, maternal care continuity	Crisis signalling: urgency and risk acknowledgement	Heightened professional solidarity; burnout risk where signals inconsistent	(31, 53)
	Digital health platforms	Smartphone tools, digital HR systems	Technological modernisation signalling	Reframing of competence around digital literacy; identity upgrading	(21, 40, 42)
	HRM investment & absenteeism	Workforce productivity reforms	Credible investment signalling improves perceived organisational support	Reduced absenteeism via strengthened reciprocity norms	(13)
	Community health volunteer (CHV) Integration	Formalising CHV role in UHC	Legitimacy signalling to informal cadres	CHVs interpret inclusion as professional recognition	(22, 23)
Post–COVID UHC consolidation (2024–2025)	National HRH capacity modelling	UHC workforce readiness	Forward-looking macro-signalling; fiscal credibility	Increased long-term career optimism if modelling tied to funded commitments	(1)
	Labour market forecasting	2,035 workforce projections	Market signalling to training institutions and investors	Education institutions recalibrate supply pipelines	(2, 3)
	Financing & budget alignment	Immunisation and NHIF reform	Financial commitment signalling	Interpretation contingent on timely disbursement consistency	(18, 19, 27)
	Gender & leadership pathways	Career inequality reforms	Equity signalling; inclusion commitments	Women health workers reassess advancement opportunities	(20)
	PPP & multi-sector collaboration	COVID PPP models	Partnership signalling across public-private divide	Reduced boundary uncertainty; collaborative identity formation	(29, 54)
	Advanced modelling & cost-effectiveness	Fiscal sustainability analyses	Technocratic signalling of reform maturity	Professional shift toward evidence-based planning mindset	(37, 55)
	Digital leadership competency	Digital management capacity	Governance-modernisation signalling	Leadership legitimacy tied to digital competence	(40, 42)

Table 2 summarises key findings from the reviewed literature mapped against the study's research question—how health-workforce features produce signals that are interpreted through local sense-making processes to shape policy, deployment and service outcomes. For each thematic area (e.g., workforce distribution, training and task-sharing, governance and financing, innovations, retention, service impacts and barriers), the table lists the primary signalling functions (measurable cues), the sense-making implications for managers, frontline workers, policymakers and communities, and key supporting references.

**Table 2:** Summary of the key findings.

Research question	Emerging thematic areas
The current landscape of HRH in Kenya across public and private health sectors?	Workforce size, distribution, and sectoral differences: (14, 40) Training, education, and task-sharing policy: (8, 41, 56) Policy and regulatory frameworks, signaling, devolution: (2, 8, 43) Funding, infrastructure, data systems, and governance: (14, 18, 19) Innovations and interventions (digital health, CHVs, task-shifting): (21–25)
Key challenges and barriers affecting the recruitment, retention, and motivation of health workers in Kenya's public and private healthcare sectors?	Retention, motivation, and job satisfaction: (50, 51, 57, 58). Funding, budgeting, and resource allocation: (1, 14, 18, 19, 50, 55). Challenges and barriers (governance, devolution, gender): (2, 14, 16, 20, 43, 44, 50, 55) Workplace conditions and infrastructure: (15, 18, 21, 59) Sector-specific dynamics and signaling: (8, 21, 50) Interventions with potential impact on retention: (8, 24, 57, 60)
Innovative interventions and strategies have been documented to improve HRH capacity, distribution, and performance in Kenya?	Retention, motivation, and job satisfaction: (1, 50, 57, 58) Funding, budgeting, and resource allocation: (1, 14, 18, 19) Organizational and governance challenges (devolution, gender, signaling): (2, 14, 20, 44) Workplace conditions and infrastructure: (15, 18, 21, 59) Signaling and private-public partnerships: (8, 21) Strengthening transformational leadership, managerial capacity, and supportive supervision (57, 58). Stabilizing funding streams, ensuring timely remuneration, and aligning county and national HRH plans (14, 18, 19). Formalizing task-sharing policies to optimize workforce use, reduce workload pressure on specialized cadres (8, 24). Investing in infrastructure, safety culture, and mental health (60–62).

Table 3 synthesises evidence from the provided reference corpus to map major health-workforce themes to their signalling functions (what measurable cues or signals each theme emits) and sense-making implications (how managers, policymakers, frontline workers and communities interpret and act on those signals). Each row links a thematic area to practical implications for decision-making and cites key references supporting the mapping.

**Table 3:** Signalling and sense-making implications of health workforce themes in Kenya.

Theme	Signalling implication	Sense-making implication	Key references
Workforce size, distribution, sectoral differences	Geospatial and labour-market data signal gaps, maldistribution and priority areas for recruitment/incentives	Local managers and planners interpret these signals to target hiring, redistribution and resource allocation	(2, 4, 5, 52)
Training, education, task-sharing policy	Presence of training programs, pre-service education and task-sharing guidelines signals capacity to expand roles and coverage	Frontline staff, supervisors and regulators use these cues to reassign tasks, adopt practices and plan continuing education	(7, 8, 15, 24, 56, 63)
Policy & regulatory frameworks, devolution	Formal policies, regulatory frameworks and devolved authority signal who has mandate, resources and accountability	Counties and institutions interpret policy signals differently, shaping implementation choices and priority setting	(2, 12, 14, 19, 43)
Funding, infrastructure, data systems, governance	Budget allocations, HRIS, facility investments and PPPs signal political commitment, operational readiness and capacity to scale	Managers, funders and communities use financial and data signals to assess reliability, plan responses and prioritise investments	(18, 29, 36, 40, 42, 55, 64)
Innovations & interventions (digital health, CHVs, task-shifting)	Deployment of apps, CHV programs, task-shifting and digital tools signal innovation, readiness and implementation intent	Users, supervisors and policymakers evaluate feasibility, trust, effectiveness and scale-up potential from these signals	(8, 21–23, 40, 65, 66)
Retention, motivation, job satisfaction	Strikes, absenteeism, low morale and workforce management investments signal systemic fragility or remediation efforts	Staff narratives and leadership interpretation determine retention strategies, incentive design and workplace reforms	(9–11, 13, 32, 50)
Funding, budgeting, resource allocation	Funding flows, budget transparency and modelling studies signal fiscal space and priorities for workforce investment	Counties and facilities interpret fiscal signals to decide hiring, training, procurement and service continuity	(1, 3, 18, 27, 37, 55)
Challenges & barriers (governance, corruption, gender, inequity)	Corruption, political interference, leadership gaps and gendered career pathways distort or mute signals and reduce credibility	Distortions lead to misinterpretation of data, misplaced priorities and misaligned decisions unless governance, accountability and equity are strengthened	(14, 16, 17, 20, 44, 62)
Service delivery impacts & community responses	Changes in workforce signals (availability, strikes, CHV activity) signal service reliability to users and influence care-seeking	Communities and patients make utilisation decisions based on perceived signals; community engagement shapes interpretation and trust	(9–11, 22, 28, 31, 38)

## 5 Discussion

The data extraction and synthesis were designed to surface the signals emitted by workforce, policy and system inputs and to examine how those signals are interpreted locally. We systematically extracted evidence from the reference corpus and organized findings into thematic categories (e.g., distribution, training, financing, governance, digital innovations). Using a signalling and sense-making lens, we interpreted how measurable signals (HRIS, budgets, geospatial analyses, strike events, digital tools) interact with institutional and community sense-making processes (leadership, devolution, community committees, frontline narratives) to produce observed outcomes. This approach foregrounds not just what the system looks like, but how actors perceive and act on available information—informing targeted recommendations to improve both signal quality and local interpretive capacity.

### 5.1 Landscape of HRH in Kenya through signalling and sensemaking lenses

During the pre-devolution period (2011–2014), HRH research in Kenya was characterised by fragmented studies that highlighted specific challenges (e.g., shortages among particular health professional cadres, uneven training opportunities) but lacked a comprehensive governance framework or systems perspective (8, 64). The absence of a coherent signalling framework—including clear national commitments, workforce deployment plans, and standardised training pathways—left frontline health workers with ambiguous career signals and uncertain prospects in the public sector. Limited visibility into how policy intentions would translate into concrete staffing, remuneration, or development opportunities contributed to early demotivation and fragmented HRH planning (64). Sensemaking theory explains how these informational gaps led workers to interpret the system as inconsistent and unpredictable, undermining trust in national guidance and anticipated career trajectories. The early devolution era (2015–2018) heightened awareness of HRH issues, particularly distribution, motivation, and shortages. Counties gained authority over health service delivery, leading to a greater focus on HRH governance at the subnational level (8, 50). Signalling theory holds that the transition to county-led health governance sent signals about accountability, resource allocation, and local hiring practices; however, these signals were not always aligned with national health priorities or the realities of workforce deployment in rural settings (8). The mismatch between facility expansion and insufficient personnel deployment created perceived capacity gaps, exacerbating workforce anxiety and increasing attrition risks in public facilities that expanded without adequate staffing (50). Sensemaking suggests that health workers and managers navigated a dynamic yet uncertain environment in which evolving governance roles, budgetary uncertainties, and inconsistent supervision affected motivation and retention, particularly in rural counties where vacancies persisted.

During the maturing devolution period (2019–2021), the literature increasingly examined HRH governance, leadership, and management. The focus shifted towards governance structures and leadership capacity, recognising that effective county-level stewardship could influence service delivery outcomes. Signalling theory emphasises the need for cohesive, consistent messaging about HRH roles, career pathways, and performance expectations across national and county governance frameworks. When signals are aligned, healthcare workers perceive a more stable and predictable career pathway within devolved governance (1, 50). Furthermore, the literature highlights how enhanced HRH governance structures—such as clear accountability and visible investments in HRH management—can transform frontline staff perceptions from uncertainty to a sense of security and professional legitimacy. The expansion of health information systems (HIS) to inform decentralised planning is also underscored as a critical signalling element for credible governance and workforce planning (50, 64).

COVID-19 spurred research on surge capacity, digital health systems, CHV resilience, and task-sharing innovations, driven by the pandemic's effects (2022–2023). Signalling theory emphasises that the pandemic intensified signals about urgent capacity expansion and digital health adoption, which could reassure workers about the system's responsiveness or cause fatigue if signals exceed actual implementation. Studies documented the rapid deployment of digital health tools, remote supervision, and increased task-shifting to meet rising demands, demonstrating a renewed commitment to adapting HRH arrangements to crisis conditions (29, 32, 57). Sensemaking shows that health workers' experiences during the pandemic—such as repeated appeals for CHV support, changes in supervision, and shifting roles—shaped new collective understandings of risk, safety, and professional identity, affecting retention based on perceived support and resource availability (14, 29, 34). The pandemic also prompted public-private partnerships (PPPs) and increased private-sector engagement in crisis response, marking a move towards diversified HRH partnerships and data-driven management, with implications for governance and workforce development (14, 16, 29).

The ongoing reform consolidation phase (2024–2025) is characterised by advanced modelling, policy experimentation, and multi-sectoral collaboration, incorporating PPPs and digital health partnerships alongside workforce professionalisation. Signalling theory suggests that this era is defined by more explicit, widely communicated commitments to HRH enhancement, standardised competencies, and cross-sector collaboration to align capacities with UHC objectives. The literature indicates that successful reforms require credible, consistent signalling on funding, career progression, and performance accountability, together with governance structures that translate these signals into predictable workplace experiences for health workers (58, 59). According to sensemaking theory, transparent implementation, visible improvements in working conditions, and tangible professional development opportunities are essential for frontline workers to view these changes as genuine system improvements rather than temporary policy adjustments (50, 60).

### 5.2 Phased evolution of signalling and sensemaking in Kenya's HRH reforms (2013–2025)

Figure 2 and Table 1, which classify interventions across reform phases, reveal a structured evolution in signalling intensity and frontline sensemaking within Kenya's HRH system. Using signalling theory and sensemaking theory as interpretive lenses, five reform phases are evident.

#### 5.2.1 Pre-devolution (limited evidence: 2013–2014)

Empirical evidence directly preceding or immediately following devolution is limited but indicates early structural disruption. Studies examining the effects of devolution on maternal health services show institutional uncertainty and service instability (12). Early strike-related disruptions highlight fragility in workforce governance and labour relations (11). Weak and fragmented. Signals regarding remuneration, deployment authority, and fiscal commitment were inconsistent across transitioning governance structures. Whereas the Sensemaking trajectory is depicted in frontline actors engaged in coping and improvisation. Professional interpretation during this period was dominated by uncertainty over employer identity, accountability lines, and job security.

#### 5.2.2 2015–2018: Service and workforce system strengthening

As devolution stabilised, reform signalling intensified around service expansion and workforce strengthening. County-level HRH priority-setting processes began institutionalising workforce planning mechanisms (14). Workforce distribution and labour market trends were more clearly quantified (2). Studies on healthcare worker motivation in public and mission hospitals demonstrate attempts to stabilise workforce morale (50). High policy signalling but variable credibility. Workforce planning tools and labour market analyses signalled reform intent, yet implementation capacity differed across counties (14). Ambiguity and role negotiation characterised this period, depicting a sensmaking trajectory. Health workers recalibrated expectations around county-level authority, performance oversight, and career progression.

#### 5.2.3 2019–2021: Governance and HRH management maturity

The literature during this phase shows increasing consolidation of governance. HRH management practices were linked to system efficiency outcomes (16). Strike impact studies highlight accountability pressures and labour-management tensions (9, 10). Workforce happiness, managerial experience, and motivation studies reflect deeper exploration of internal organisational climate (32, 51). Health research governance analyses also signal institutional strengthening (39) based on the intervention identifed over the years. Performance and governance signalling strengthened. Accountability, managerial competence, and data use became central reform messages (16). The sensemaking trajectory is depicted in managerial accountability, which has become internalised. Health workers increasingly interpreted reforms through the lenses of performance metrics, professional responsibility, and organisational culture rather than structural instability.

#### 5.2.4 2022–2023: Digital acceleration and crisis adaptation

The COVID-19 period generated crisis signalling and rapid system adaptation. Health facility readiness and Infection Prevention and Control (IPC) preparedness studies demonstrate the urgency of reform communication (53). PPPs to scale the COVID-19 response reinforced signals of collaborative governance (29). Investments in HRM&D and data use were correlated with reductions in absenteeism, indicating performance-digital integration (13). Mental health literacy among primary health care workers reflects the expansion of psychosocial capacity (26). Task-sharing policy institutionalisation strengthened scope-of-practice signalling (8). Additionally, informal-sector engagement with the National Hospital Insurance Fund (NHIF) highlights challenges to system inclusion during UHC expansion (27). Crisis signalling was strong and urgent, combined with digital and partnership narratives. Reform messages emphasised resilience, adaptability, and integration. Crisis solidarity emerged alongside digital identity reconfiguration as a sense-making trajectory. Health workers adapted to rapid task-sharing, data reporting expectations, and new accountability frameworks.

#### 5.2.5 2024–2025: UHC consolidation, modelling, PPPs, and professionalisation

The most recent phase demonstrates macro-level strategic signalling aligned with UHC consolidation. National HRH capacity assessments signal workforce sufficiency benchmarking (1). Advanced health labour market modelling projects long-term supply-demand scenarios through 2035, thereby signalling forward-looking fiscal credibility (3). Gender and leadership equity studies signal professionalisation and career pathway reform (20). Immunisation financing analyses highlight fiscal bottlenecks under decentralised governance (18). HRH management efficiency analyses further consolidate governance maturity (16). Budget space modelling for workforce investment reinforces macro-fiscal sustainability narratives (55). Digital transformation readiness and management competency development studies support strategic digital integration under UHC (40, 42). Strategic macro-signalling is strongest in this phase. Signals emphasise fiscal sustainability, modelling credibility, workforce forecasting, and structured professional pathways. Professionalisation and strategic career recalibration dominate. Health workers increasingly interpret reforms as long-term institutional commitments rather than episodic or crisis-driven changes.

### 5.3 Key challenges and barriers

#### 5.3.1 Workforce size and distribution

The literature consistently documents an uneven distribution of health workers, with a concentration in urban areas and understaffing in public facilities serving rural populations (14, 40). This pattern aligns with broader HRH concerns, indicating that public sector staffing remains fragmented, potentially signalling a weaker commitment to rural service delivery and discouraging workforce retention in underserved areas (43). The implications for UHC are clear: without equitable geographic distribution, the health system can signal to communities that coverage is uneven, undermining trust and utilisation (14, 40).

#### 5.3.2 Training, education, and skills development

Training systems in Kenya vary in quality, standardisation, and relevance to evolving service needs (e.g., digital health, maternal and child health priorities) (41, 56). This aligns with dynamics in which health workers perceive training opportunities as inconsistent with frontline demands, reducing motivation to remain in or enter the public sector (44). Task-sharing policies have been implemented to optimise skill use and expand service delivery, signalling adaptability; however, effective implementation remains inconsistent across counties (8). Reviews highlighting gaps between training and workforce needs support the need for cohesive competency frameworks (56).

#### 5.3.3 Policy and regulatory frameworks

Kenya's HRH policy environment includes devolved governance, task-sharing guidelines, and efforts to standardize scopes of practice; however, implementation fidelity is variable, producing mixed messages to the workforce about career prospects and security (8, 43). This aligns with the premise that inconsistent policy messages can erode perceptions of career pathways and job security, thereby affecting recruitment and retention decisions (43).

#### 5.3.4 Sector-specific challenges and data systems

In both the public and private sectors, persistent funding constraints, wage delays, and inefficient resource allocation are repeatedly cited as barriers to stable staffing and motivation (14, 18, 19). Deficits in workplace infrastructure and supply bottlenecks further exacerbate job dissatisfaction and absenteeism, reinforcing perceptions of an unsupportive work environment (18). The literature notes that private facilities often maintain higher staffing levels and better distribution than public facilities, highlighting sectoral divergence in HRH signalling and its consequences for workforce morale (21, 24).

#### 5.3.5 Retention, motivation, and job satisfaction

Dissatisfaction and demotivation across the public and private sectors stem from low and irregular remuneration, limited professional development, and weak recognition, contributing to higher turnover intentions among health workers (1, 50). Transformational leadership and supportive management climates appear to bolster job attitudes and retention by enhancing perceptions of organisational support and career progression potential (57, 58). The private sector often offers more attractive conditions, intensifying competition for skilled personnel and pressuring public facilities to address morale and retention to maintain service delivery (50). These dynamics align with broader evidence that job satisfaction is a central predictor of retention in resource-constrained health systems (57, 58).

#### 5.3.6 Funding, budgeting, and resource allocation

Chronic underfunding, delayed wage disbursements, and erratic budget cycles undermine workforce stability and motivation. Kenya's health sector faces recurrent gaps in adequate financing for salaries, allowances, and workforce development, with delays in county-level budget execution exacerbating uncertainty and demotivation among staff (14, 18, 19). Inadequate funding constrains hiring, training, supervision, and retention strategies, creating instability that contributes to absenteeism and turnover in both public and private settings (14, 18, 19). These financial limitations are consistently identified as foundational barriers to HRH performance and continuity of care (1, 14, 17).

#### 5.3.7 Organisational and governance dimensions

Devolution has fragmented HRH governance, creating ambiguities over national vs. county roles and complicating recruitment planning, salary administration, and career progression signals. This governance ambiguity creates an unstable environment for workforce planning and can deter long-term commitment, particularly among cadres with mobility options to the private sector (2, 16, 44). Political interference, patronage, and perceived inequities in hiring practices further erode trust and willingness to participate in public-sector employment, thereby contributing to retention challenges in underserved regions (2, 14, 44). Gender disparities and limited opportunities for women's advancement also constrain recruitment and progression, underscoring the need for equity-focused HRH policies (20, 44).

#### 5.3.8 Workplace conditions and infrastructure

Working environments in many public facilities are hampered by insufficient infrastructure, stockouts, inadequate sanitation, and overcrowding, which reduce job satisfaction and increase stress, burnout, and intentions to exit the public sector. The absence of essential supplies, combined with high patient loads, creates a work environment that many staff find unsustainable, contributing to attrition and reduced motivation in both public and private facilities when competitive alternatives exist (15, 18, 21). In rural and peri-urban settings, professional isolation and limited peer support exacerbate morale problems, adversely affecting retention decisions (59).

#### 5.3.9 Sector-specific dynamics and signalling

Private facilities, while often better staffed and resourced, intensify competition for skilled personnel, creating a bifurcated labour market in which public-sector roles may appear less attractive. This signalling effect can drive talent away from public facilities, undermining equity of access to care in underserved areas and complicating the achievement of UHC goals (21, 50). Conversely, improvements in public-sector salary regimes, signing incentives, and clear career ladders, when paired with reliable funding and transparent governance, can enhance retention (8).

### 5.4 Evidenced innovative interventions and strategies

#### 5.4.1 Digital health solutions and data-enabled management

Documented innovations in mobile and digital tools have been used to support health workers' performance, data management, adherence to guidelines, and remote training. Examples include health worker–targeted smartphone apps for clinical decision support and malaria RDT quality assurance, as well as mHealth messaging to caregivers and support for CHV activities (8, 21, 24). These interventions signal a modernisation of HRH practice and can improve efficiency and data quality when well implemented (52, 65).

Critical to signalling and sensemaking are positive signals around digital adoption (investment, training, interoperability), which can boost health workers' perceived competence and organisational support, enhancing motivation and retention. Conversely, evidence of weak infrastructure, inconsistent roll-out, or poor data governance sends signals of instability and potential retrenchment, which may erode trust and dampen adoption among frontline workers (52, 65, 66). Public Health Technology Assessment (HTA) literature emphasises evaluating not just feasibility but also value, equity, and potential harms (e.g., data privacy concerns, increased workload, or marginal benefits) to avoid unintended consequences that could undermine HRH performance (63, 66, 67).

Sustainability challenges arise when digital health initiatives depend on project-specific funding, external partners, or short-term pilots. Sustainability requires local investment, strong governance, interoperability, capacity-building (training in digital health literacy and data governance), and integration with existing HMIS frameworks to avoid data silos and discontinuity post-donor support (54, 66, 68). The literature on digital health literacy further emphasises that interventions must address user training, trust, privacy, and accessibility to avoid widening inequities in service delivery (53, 69).

#### 5.4.2 Task-shifting and workforce optimisation

Task-shifting policies and guidelines have been pursued to extend healthcare service delivery in Kenya by reallocating duties across health worker cadres, accompanied by training and regulatory clarifications (63, 68). Health workers can interpret this approach as an increased opportunity to apply skills and develop, thereby enhancing motivation and job satisfaction when coupled with appropriate supervision and compensation (8, 54, 63). Evidence from studies suggests that task-shifting can improve service access when implemented with quality assurance, workforce planning, and continuous training (63, 68).

Clear task-shifting policies signal organizational commitment to adapt to workforce shortages and to harness the capacity of non-physician cadres. When accompanied by standardized training and supervision, this improves workers' sensemaking about their roles and career trajectories, potentially boosting retention in high-demand cadres. If signalling is inconsistent or poorly implemented, health workers may perceive greater role ambiguity and job insecurity, reducing motivation and adherence to new duties (1, 8).

#### 5.4.3 Community health system strengthening and CHV engagement

Strengthening CHV training and integrating CHVs into formal supervision and referral networks have been recognized as effective strategies to improve maternal and child health outcomes, reinforcing the value placed on primary care and community-based delivery (66). Such strategy can enhance sensemaking among frontline workers by emphasizing their essential role in health systems, potentially improving retention in rural and underserved areas (66).

#### 5.4.4 Public–private collaboration and health system resilience

PPPs and private-sector involvement in HRH capacity-building are noted as mechanisms to accelerate HRH strengthening, signal cross-sectoral commitment, and leverage resources during health emergencies (54, 69). Effective PPPs provide indications of sustainability and strategic alignment with UHC goals, which can positively influence health workers' perceptions of career stability and growth opportunities within a more diversified health system (54, 69).

#### 5.4.5 Leadership development and governance reforms

Investment in leadership development and governance reforms is critical for translating HRH investments into improved performance. Studies emphasize the role of transformational leadership in shaping job attitudes, reducing burnout, and fostering an enabling work environment, thus contributing to better recruitment and retention outcomes (53, 67). Evidence suggests that leadership training and governance capacity-building signal institutional commitment to staff welfare and professional advancement (53, 67).

#### 5.4.6 Training, mentoring, and capacity-building ecosystems

Capacity-building approaches—ranging from on-the-job training and mentorship to continuous professional development—have been associated with improved performance and retention when embedded in regular supervision and aligned with local health priorities (70, 71). These interventions reinforce positive signals of ongoing investment in staff skills, supporting motivation and a sense of professional belonging (70).

#### 5.4.7 Health system performance and health outcomes

Interventions targeting HRH capacity and performance—through digital health, task sharing, CHV integration, and leadership/governance reforms—are linked to improved service delivery and health outcomes in various contexts. Although Kenya-specific outcomes can vary, broader literature indicates that well-designed HRH interventions can improve service utilisation and patient satisfaction (70, 71). The successful outcomes reinforce positive sensemaking and ongoing engagement among health workers (53, 67, 72).

### 5.5 Signalling and sense-making: interpreting HRH reforms to inform policy and practice

This study applies the signalling and sense-making lens across Kenya's health-system transition—from pre-devolution arrangements, through devolution implementation, to current efforts at UHC consolidation—to explain how workforce signals and local interpretive capacities have changed and influenced outcomes. In the pre-devolution period, centralised HR policies and limited local data produced weak, delayed signals that constrained responsive staffing and training (14). Devolution redistributed authority and created new subnational signalling environments: counties assumed responsibility for HR decisions but often lacked robust HRIS, clarity on financing, and managerial capacity to interpret and act on workforce signals, leading to variable implementation and priority-setting across counties (2, 14). As Kenya moves toward UHC consolidation, the combination of improved signalling (geospatial and labour-market analyses, HRIS, targeted investments and digital pilots) and strengthened sense-making (managerial training, data use, community engagement) appears critical to translating workforce inputs into equitable coverage gains (13, 21, 55). Conversely, persistent distortions—opaque budgets, corruption, strikes, and gendered leadership barriers—continue to mute or misdirect signals, undermining UHC objectives unless governance and interpretive capacity are addressed (11, 20). Thus, our categorisation of the evidence across these stages highlights that progress toward UHC depends not only on the presence of workforce signals but on concurrent investments in the systems and actors that make sense of those signals.

The convergence of signalling and sensemaking perspectives clarifies why, even when innovations (digital tools, task sharing, community engagement) are documented, the absence of consistent policy signalling, adequate funding, and reliable data systems may disincentivise health workers to remain in or join the public sector. This underscores the need for coherent, well-communicated HRH strategies that couple investment with transparent deployment plans, performance incentives, and comprehensive data to monitor progress toward UHC goals (14, 43). The existing body of Kenyan HRH literature supports a phased approach: (a) standardize training and competency expectations; (b) implement stable financing and timely remuneration; (c) scale successful HRH interventions (digital health, task sharing, CHVs) with rigorous monitoring; and (d) strengthen county-level HRH information systems to improve governance and accountability (8, 41, 44).

## 6 Conclusion

This study examined HRH reforms in Kenya's devolved health system and highlighted several persistent challenges affecting workforce performance and stability. Key barriers identified include uneven workforce distribution across counties, inconsistent implementation of HRH policies, limited supervision and support systems, and persistent resource constraints affecting recruitment, training, and retention. These structural and governance challenges continue to shape how health workers experience and interpret HRH reforms within the devolved system.

At the same time, the findings reveal important innovations and adaptive responses that have emerged within Kenya's health sector. These include strengthened county-level workforce planning, new approaches to training and capacity building, and collaborative mechanisms between national and county governments to improve HRH coordination. Such innovations demonstrate the potential of decentralised governance to generate context-specific solutions to workforce management challenges. Drawing on signalling theory, the study further shows that HRH reforms function not only as technical policy interventions but also as signals that health workers interpret through ongoing sensemaking processes. Policies related to recruitment, remuneration, deployment, and professional development signal organisational priorities, fairness, and institutional commitment to the workforce. When these signals are clear, credible, and consistently implemented, they can strengthen workforce engagement and motivation. Conversely, inconsistent or poorly communicated reforms may generate uncertainty and weaken trust within the health workforce. Overall, the study underscores that strengthening HRH systems in devolved contexts requires attention to both technical policy design and the organisational signals these policies convey to health workers. Addressing the identified barriers as emerging innovations scale can help improve workforce motivation, retention, and performance. These findings provide practical lessons for Kenya and other low- and middle-income countries pursuing UHC through decentralised health governance.

## Data Availability

The original contributions presented in the study are included in the article/Supplementary Material, further inquiries can be directed to the corresponding author.
